# Educational intervention program based on health belief model and neck pain prevention behaviors in school teachers in Tehran

**DOI:** 10.1186/s12889-022-13873-8

**Published:** 2022-08-06

**Authors:** Zohreh Moradi, Sedigheh Sadat Tavafian, Seyedeh Somayeh Kazemi

**Affiliations:** 1 Department of Health Education and Health Promotion, Faculty of Medical Sciences, Tarbiat Modares University, Tehran, Iran; 2 Department of Health Education and Health Promotion, Tarbiat Modares University, Tehran, Iran; 3 Department of Public Health, School of Health, Mazandaran University of Medical Sciences, Sari, Iran

**Keywords:** Occupational neck pain, Teachers, Educational intervention, Health belief model

## Abstract

**Background:**

Prevention of musculoskeletal disorders as one of the most common occupational health problems among the working population in both developed and developing countries is an important necessity and priority. The aim of this study was to evaluate the effectiveness of an educational intervention program based on the Health Belief Model (HBM) to increase awareness, perceived sensitivity, perceived severity, perceived benefits, and self-efficacy in adopting neck health-promoting behaviors in school teachers**.**

**Methods:**

The present study was a quasi-experimental of the randomized clinical trial that was conducted for 6 months (December 2020 to July 2021). Participants were 146 junior high school teachers were selected from 26 schools through random sampling and divided into two groups of intervention and control. The data collection instrument was the self-design questionnaire and was completed in three points of time (before, immediately, and 3 months after the intervention). The data were analyzed by software version 24 SPSS.

**Results:**

The results showed that awareness, perceived sensitivity, perceived severity, perceived benefits and barriers, and self-efficacy in adopting neck health-promoting behaviors in the intervention group increased in two points of time (immediately after the intervention and 3 months of follow-up) (*P* <  0.05).

**Conclusion:**

Designing and implementing an educational intervention based on HBM could affect in adopting neck health-promoting behaviors among teachers.

**Trial registration:**

IRCT20210301050542N1, 16/03/2021 first registration has been approved in Iranian Registry of Clinical Trials at (16/03/2021).

## Background

Neck pain (NP) refers to one of the most common types of work-related musculoskeletal disorders (WMSDs), which despite advances in technology is still one of the most common occupational health problems among working populations in developed and developing countries [1, 2]. These disorders can progress from mild to severe [3] and have important socio-economic consequences such as reduced productivity, early leave and retirement [4], absenteeism and imposition of medical expenses [5]. Prevalence of neck pain among different occupations accounts for about 44 to 62% of injuries [2, 6–9]. Numerous studies show that neck pain is more common among teachers than other occupations [7, 9, 10]. Statistics show the prevalence of neck pain among teachers is about 39 to 95% [3, 4, 6, 11]. The prevalence of neck pain among Iranian teachers is about 57.8% [2].

Various factors such as demographic factors (age, sex, body mass index) [12, 13], physical factors (duration of employment, inappropriate physical posture at work, excessive computer use, sitting and prolonged standing, excessive bending of the neck forward or backward, unprincipled exercise, lack of adequate rest time) [1, 2, 5, 8, 14, 15]. Psychological factors (high workload, general health, work-related stress, poor mood, lack of co-worker support, marital and family relationships, job dissatisfaction, monotonous work, organizational characteristics and financial and social aspects) in the prevalence of pain the neck plays a role in teachers [16–18]. According to studies, most of the stated causes of job-related neck pain in teachers are behavioral causes [2, 19].

There are various reasons for not performing neck health-promoting behaviors, the main reason being the lack of belief in the extent of the disease and the severity of the damage caused by the disease (perceived sensitivity and severity) and also the lack of evaluation of the benefits and barriers to health behavior (perceived benefits and barriers) [10]. Education plays is a vital role in improving people’s health and is one of the basic pillars of changing inappropriate behaviors. Proper training and regular training programs, measuring awareness and attitude, perceived sensitivity and severity, perceived benefits and barriers and self-efficacy of the target population and explaining the effective elements in the educational process can be important factors in changing behavior and improving health [20].

Research shows that the most effective training programs are based on theory/model-based approaches that are rooted in behavioral change patterns. Theories are useful for educational designers because it offers special aspects for educational interventions [20, 21]. So, choosing a health education model is the first step in the planning process of an educational program. One of the models that is used frequently associated in behavioral science studies related to health, is the Health Belief Model (HBM). The health belief model is an effective framework for designing educational interventions and promoting preventive behaviors act and considers behavior as a function of the individual’s knowledge and attitude [21, 22]. This model is evaluated by understanding factors such as perceived intensity and sensitivity, perceived benefits and barriers, and self-efficacy. According to it, a persons’ behavior changes when he understands the level of danger that threatens him (perceived sensitivity and severity) and also has a proper assessment of health barriers and behaviors (perceived barriers and benefits )[10–20]. According to the efficiency of the health belief model in different studies for prevent dangerous behaviors and promote healthy behaviors, because so far, this model has not been used to promote neck health-promoting behaviors in Iranian teachers, the aim of this study was to assessment the effect of the educational intervention program based on health belief model in adopting neck pain prevention behaviors in junior high school teachers in the 19th district of Tehran.

## Methods

### Participants

The present study was a quasi - experimental randomized clinical trial adopted from the declaration of Helsinki and received ethical approval from the Human Ethics Committee at the University of Tarbiat Modares, Tehran, Iran (IR.MODARES.REC.1399.163). The present study has been recorded in Iranian Registry of Clinical Trials (IRCT20210301050542N1), (16/03/2021). This study was conducted for 6 months from 21 December 2020 22 July 2021. After coordination with the principals and officials of the ministry of education and school principals in Tehran’s 19th district, junior high school teachers were invited to study through social media, by sending a call message and explaining the benefits of research. Out of 26 junior high schools, 220 teachers announced their readiness to participate in the research. Inclusion criteria include internet access, mobile phone, and its use skills, exclusion criteria include unwillingness to continue participating in research, having a second job, congenital musculoskeletal disorders related to the neck, history of surgery or neck vertebral fractures and medical prohibition on doing sports. A number of teachers were excluded from the study and 146 participants (mean age 38.5; standard deviation 6.5 years and mean Work experience 12.04; standard deviation 6.2) were invited to study.

The sample size was estimated with the formula of estimating the rate of 10% shedding in 120 similar studies and sampling was performed based on simple randomization method [9, 10, 20, 23]. Of all participants 119 individuals (81.51%) were female, 27 individuals (18.49%) were male, 89 individuals (60.96%) experienced neck pain and 57 individuals (39.04%) did not experience neck pain. Then, considering the 95% confidence level and 85% test power and using simple random sampling method, the participants were divided into two groups, the intervention group with 73 participants and the control group with 73 participants. The present study was three-sided blind, participants, care providers and those who evaluated the results were blind in the intervention. All participants signed an informed consent form and the study procedures were approved by the Ministry of Education in the districts where the schools were located. Table 1 shows the rest demographic characteristics of the studied participants.

**Table 1:** The characteristics of participants (*n* = 146)

Variable	No (%)
Gender	
Female	119 (81.51)
Male	27 (18.49)
Marital status	
Single	36 (24.61)
Married	110 (75.34)
Level of Education	
Bachelor	86 (58.91)
Master	53 (36.30)
Ph. D	7 (4.79)
BMI	
Normal weight(18.5–24.9)	63 (44.46)
Overweight (25–29.9)	59 (40.41)
Obese (≥ 30)	24 (16.43)
Experience of pain	
Yes	89 (60.96)
No	57 (39.04)

### Procedure

The present study was performed in three stages: pre-intervention stage, intervention stage and post-intervention stage. In the post-intervention stage, two evaluations were performed immediately after the intervention and 3 months after the intervention to follow up the effect of the intervention on the intervention group. In the pre-intervention stage, using a self-designed questionnaire based on the Health Belief Model, demographic information as well as the level of awareness, perceived sensitivity, perceived severity, perceived benefits and barriers, participants’ self-efficacy in performing health-promoting behaviors Neck, collected. Then, based on the analysis of information obtained, participants entered the intervention stage, which lasted for 4 weeks. The intervention group received the training intervention while the control group did not receive any training program. After the intervention, two post-tests were performed using the previous questionnaire, one immediately after the intervention and one three months after the intervention, from both control and intervention groups. The obtained data were analyzed and evaluated in three points of time, before the intervention (T1), immediately after the intervention (T2) and 3 months after the intervention (T3).

### Instruments

In this study, a researcher-made questionnaire based on the health belief model was used to collect data in three points of time. This questionnaire consisted of two parts. The first part consisted of demographic information and had 18 items and the second part had 8 areas and 43 questions that included: awareness (5 questions), perceived sensitivity (6 questions), perceived severity (5 questions), perceived benefits (questions), perceived barriers (4 questions), cues to action (3 questions), self-efficacy (6 questions) and behavior (9 questions). For questions in the field of awareness of the 3-part Likert spectrum, it is wrong (score 0), No idea (score 1), true (score 2). For domain questions (perceived sensitivity, perceived severity, perceived barriers, perceived benefits, self-efficacy, Cues to Action) questions in the form of a 5-point Likert scale, (completely agree 5), (agree 4), (No idea 3), (Disagree 2) and (completely disagree 5) were considered. In the field of behavior, the questions were considered based on a 5-part Likert scale (never 1), (rarely 2), (sometimes 3), (often 4), (always 5). The minimum score for neck pain prevention behaviors was 9 and the maximum score was 45.

The questionnaire was designed based on the structures of the Health Belief Model and was evaluated by the participants and experts of the research team in two stages in terms of validity, reliability and psychometrics of the structure. In this way, the questionnaire was given to 15 specialists in health education and health promotion, ergonomics, occupational health and physiotherapy to be examined in terms of appearance and content. The opinions of these people led to the correction or change of some of the questions in the questionnaire.

To calculate the reliability, the reliability assessment method was used with internal consistency method (Cronbach’s alpha) and the in-class reliability assessment was used. Cronbach’s alpha for the whole scale was (0.87) and the internal correlation coefficient was (ICC)(0.92) The section enjoys. In the external reliability of the questionnaire, which was performed by retesting, the questionnaire was sent to 30 teachers in two stages with an interval of 2 weeks. In the second stage to evaluate the validity of the structure, confirmed factor analysis and scale correlation matrix were used. After confirming the adequacy of sampling based on KMO statistics and Bartlett sphericity test (KMO = 0.833, χ2 = 5030.743 and *p* < .001), factor analysis was performed with 146 participants. Eight final factors with 43 questions were extracted from confirmed factor analysis.

The data obtained from completing the first and second stage questionnaires were measured using SPSS software version 24 and Pearson correlation which was 0.92 which showed that the questionnaire has scientific validity for use in similar studies. After the necessary explanations about the objectives of the research, how to complete the questionnaire and gain the trust of the participants in the research regarding the confidentiality of information and also their satisfaction, the questionnaire was provided to the research participants. To avoid bias, the questionnaire it was coded and provided to the participants online by someone other than the researcher. After completing the questionnaire and analyzing the results obtained from the first stage, the educational intervention was designed based on the pattern of health belief and preventive behaviors of occupational neck pain.

### Interventions

**Fig. 1:**
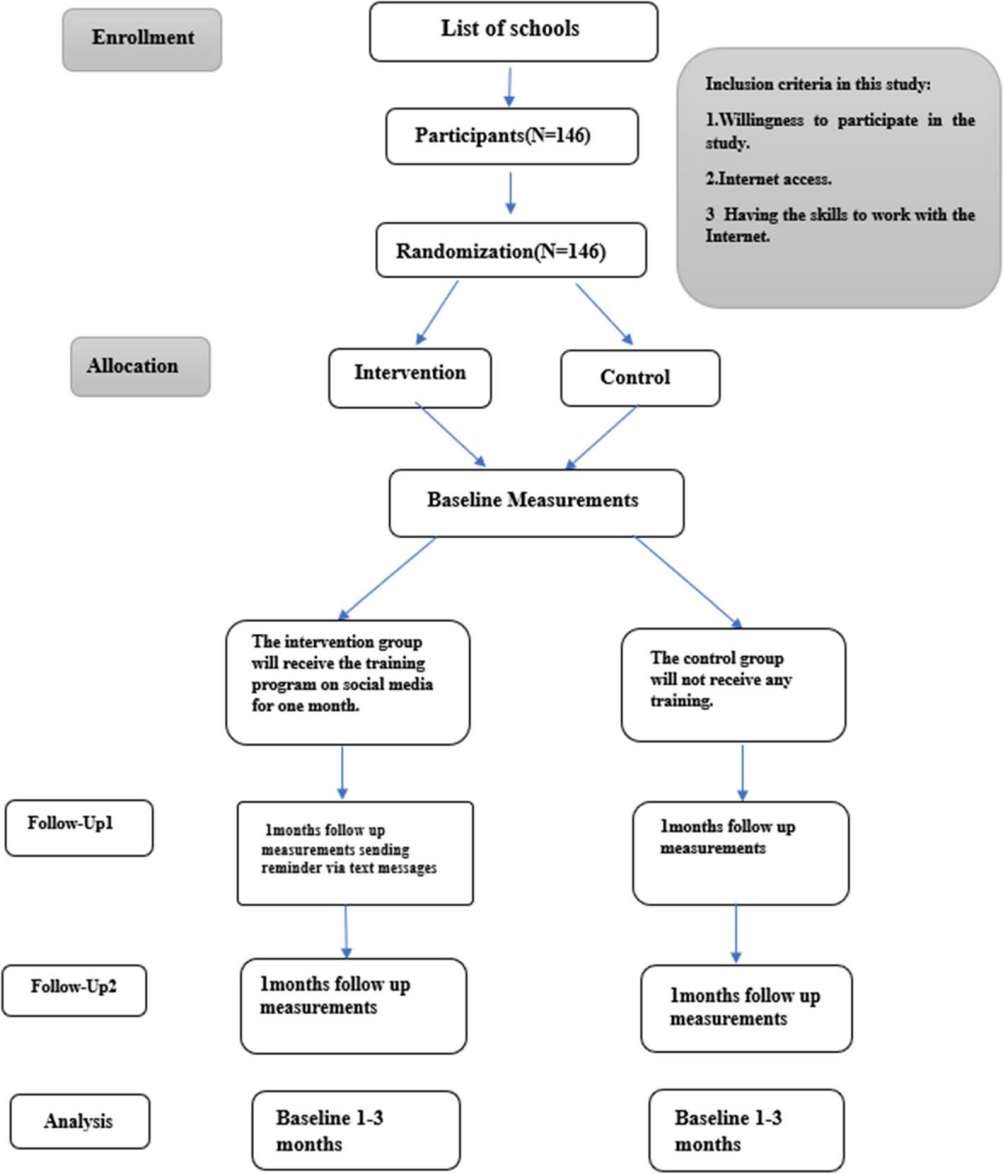
Consort flow diagram

The interventions were performed in several stages over a period of 4 weeks in the context of social networks. The first stage included holding two specialized webinars lasting 1 h with the presence of health education and health promotion specialists, ergonomics specialists and psychologists. The educational content in these webinars was included neck pain, behavioral causes of occupational neck pain, susceptible people, physical and psychological factors that cause neck pain, neck health-promoting behaviors, and ergonomic training on how to improve their posture such as correct sitting and standing, proper use of computer and mobile phone and training on how to change your workstation by changing Chair and table height, back slope, keyboard slope and location, screen height, forearm and footrest if needed., The proper way to sleep, and to do the right exercises, as well as the effect of stress and lack of healthy social communication around neck pain, as well as ways to control stress and anxiety caused by work and how to establish healthy social communication were discussed by experts. All teachings on the principles of ergonomics have been confirmed by other studies [1, 24–28]. In the next stages, educational contents include: the effect of neck pain on quality of life and work (perceived severity), benefits of neck pain prevention in teachers (perceived benefits), barriers to correct behaviors and providing appropriate solutions to control and Elimination of barriers (perceived barriers), self-efficacy skills (self-efficacy), skills and behaviors that prevent and reduce neck pain, sports movements (stretching and strengthening neck muscles) to reduce and prevent neck pain, the correct way of ergonomics in Performing activities, stress management in reducing and preventing neck pain, establishing healthy social communication (behavior) in various formats including posters, pamphlets, infographics, health text messages, podcasts, animations and videos on a daily basis for the intervention group it placed. Also, once a week, question and answer sessions were held in the presence of experts and participants to answer the questions and remove the ambiguity of the participants regarding the educational contents in the context of the social network. To participate in training sessions by calling each of the participants and mentioning the time and the duration of attending the class was coordinated with them. No educational intervention was performed for the control group during this period. Immediately after completing the educational interventions, the questionnaire was used again on the basis of the codes assigned to each person in the first step and to evaluate the effectiveness of training provided to study participants and relevant information was collected. During this period no educational intervention was performed for the control group. After done necessary interventions to evaluate the consolidation of the training provided for 3 months both groups were given opportunities. During this period, in order to remind the educational contents, educational materials were provided to the intervention group twice a week in the context of social networks, and once or twice a month, telephone calls were made to each member of the intervention group and the necessary items were given to them. After 3 months, the research participants were invited again the questionnaire was given to them and after completion questionnaires were collected and the obtained data were analyzed. Figure 1 shows the intervention steps.

### Statistical analysis

The collected data were analyzed using SPSS24 software. Shapiro-Wilk and Skewness tests were used to evaluate the normality of the data. One-way repeatable ANOVA test with Bonferroni was used to compare the changes in each group (in three time periods). Independent t-test was used to compare the mean of quantitative data between the intervention and control groups. Chi-square test and Pearson correlation were used to compare the frequency of qualitative data between the intervention and control groups (before, immediately after and 3 months after the intervention).

## Results

The study participants were mostly women. Accordingly, 81.51% of the total population was female and 18.49% were male. Most of the participants in the study were married. The rest characteristics of whole participants were shown in Table 1 .The mean age of the intervention group was (37.6 ± 6) and the control age group was (39 ± 7) years. Statistical analysis showed that no significant differences were observed between the variables of the intervention and control groups (Tables 2, 3).

**Table 2:** Demographic characteristics of participants (based on quantitative variables) in intervention and control groups

Variable	Intervention group(*N* = 73) (Mean ± SD)	Control group(*N* = 73) (Mean ± SD)	(*P* value)a
Age	37.6 ± 6	39 ± 7	0.763
Height	165 ± 8	168 ± 4	0.731
Weight	70 ± 10	73 ± 9.1	0.963
Body mass index	26 ± 3	25 ± 3	0.952
Number of children	0 ± 1	1 ± 1	0.524
Work experience	12 ± 7	14 ± 8	0.693
Cigarettes (No)	73 ± 1.2	73 ± 2.9	0.596
Sports activities			
No exercise	58 ± 2.3	48 ± 3.2	0.671
≤ 3 days a week	9 ± 1.3	14 ± 4.1	0.527
> 3 days a week	6 ± 1.1	11 ± 3.6	0.541

a. Independent T-test

**Table 3:** Demographic characteristics of participants (based on qualitative variables) in intervention and control groups

Variable	Intervention group(*N* = 73)	Control group(*N* = 73)	(*P* value)a
Gender			
Female	65 (89)	52 (71)	0.573
Male	8 (11)	21 (29)	0.612
Marital status			
Single	17 (24)	19 (27)	0.685
Married	56 (76)	54 (73)	0.575
Level of Education			
Bachelor	48 (65)	38 (51)	0.499
Masters	25 (35)	35 (49)	0.642
Housing situation			
Rental	27 (36)	18 (23)	0.783
Owner	46 (63)	52 (71)	0.651
Employment Status			
Contractual	34 (46)	16 (20)	0.652
Official	39 (54)	57 (76)	0.902
The economic situation			
≤ 1000$	23 (31.5)	14 (19.17)	0.496
> 1000$	50 (68.5)	59 (80.83)	0.518

a. Chi-square test

The knowledge score in both groups before the intervention was not significantly different (*p* = 0.063), while after the intervention this score in the intervention group was higher than the control group (*p* = 0.002). Furthermore, before the intervention there was no statistically significant difference between the two groups in terms of perceived sensitivity (*p* = 0.085), but after the intervention, this difference was statistically significant (*p* = 0.001) (Table 4).

**Table 4:** Pre- and post-intervention comparative results in the intervention and control groups

Variable	Intervention group(*N* = 73) (Mean ± SD)	Control group(*N* = 73) (Mean ± SD)	(*P* value)a
Knowledge			
T1	7 ± 1	7.07 ± 2	0.063
T2	9.07 ± 1.01	6 ± 1	0.002
T3	9.8 ± 1.087	5 ± 1.04	0.002
Perceived sensitivity			
T1	23 ± 3.073	20 ± 3	0.085
T2	25 ± 2	19 ± 2.8	0.001
T3	26 ± 2	17 ± 2.04	0.001
Severely perceived			
T1	19.01 ± 2	17 ± 4	0.073
T2	20 ± 2	16 ± 4	0.001
T3	21 ± 2	15 ± 4	0.001
Perceived benefits			
T1	20 ± 2.016	18 ± 4	0.437
T2	21 ± 2.036	17 ± 4.041	0.002
T3	21 ± 2.02	16 ± 4	0.001
Perceived obstacles			
T1	17 ± 3.064	12 ± 3	0.093
T2	16 ± 2	13.1 ± 3	0.025
T3	14 ± 1.01	14 ± 3	0.013
Cues to Action			
T1	10 ± 0.87	10 ± 3.2	0.071
T2	12 ± 0.76	10 ± 3	0.011
T3	13 ± 0.42	8 ± 3	0.001
Efficacy			
T1	21 ± 3	20 ± 4.01	0.083
T2	25 ± 2	19 ± 4	< 0.001
T3	26 ± 2	16 ± 4	< 0.001
Behavior			
T1	27 ± 5	23 ± 5	0.052
T2	39 ± 5	21 ± 4	< 0.001
T3	39 ± 5	20 ± 3.6	< 0.001

a. Independent T-test

Before the educational intervention, the mean perceived intensity in the two groups was not significantly different (*p* = 0.073), but this difference immediately and 3 months after the intervention was statistically significant (*p* = 0.001) (Table 4). Moreover, in spite of being the same groups regarding perceived benefits before the intervention (*p* = 0.437), but they were statistically different immediately and 3 months after the (*p* = 0.001) (Table 4). Regarding perceived barriers there was no significant difference between both groups before the intervention (*p* = 0.093), but immediately and 3 months after the intervention this difference was statistically significant (*p* = 0.013) (Table 4).

Cues to Action score before the intervention in the two groups did not show a significant difference (*p* = 0.093), but after the intervention the two groups had a statistically difference, in this regard (*p* = 0.001) (Table 4). In terms of self-efficacy mean score there was no difference between the two groups before the intervention but after the educational intervention this difference was significant (*p* <  0.001) (Table 4).

Finally, regarding the average score of neck health-promoting behaviors the results showed there was no statistically significant difference before the intervention (*p* = 0.052) but after the intervention, this difference was statistically different (*p* <  0.001) (Table 4).

The results of the educational intervention showed that the number of people who had experienced neck pain before the educational intervention decreased from 89 individuals (60.96%) to 41individuals (28.08%). Comparative results before and after the intervention based on (One-way repeatable ANOVA test) in the intervention and control groups was shown in Table 5.

**Table 5:** Comparative results before and after the intervention in the intervention and control groups

Variable	(***P*** value before and after)a	
Knowledge	df = 2 f = 19.45 *p* < 0.001	df = 2 f = 4.38 *p* = 0.052
Perceived sensitivity	df = 2 f = 13.71 *p* = 0.002	df = 2 f = 26 *p* = 0.061
Severely perceived	df = 2 f = 17.22 *p* < 0.001	df = 2 f = 20.41 *p* = 0.084
Perceived benefits	df = 2 f = 13.3 *p* = 0.001	df = 2 f = 15.26 *p* = 0.052
Perceived obstacles	df = 2 f = 8.33 *p* < 0.001	df = 2 f = 0.29 *p* = 0.061
Cues to Action	df = 2 f = 0.29 *p* < 0.001	df = 2 f = 5.53 *p* = 0.072
Efficacy	df = 2 f = 12.57 *p* < 0.001	df = 2 f = 29.87 *p* = 0.051
Behavior	df = 2 f = 13.1 *p* < 0.001	df = 2 f = 21.12 *p* = 1

aOne-way repeatable ANOVA test

## Discussion and conclusions

Neck pain is one of the most common musculoskeletal disorders, teachers due to the nature and context of work and job responsibilities, are exposed to various factors that threaten the health of the neck [6, 11]. The aim of this study was to investigate the effect of educational intervention based on the health belief model on the adoption of neck pain prevention behaviors on teachers in social networks. Based on the findings of this study, after the educational intervention, the mean score of awareness, perceived sensitivity, perceived severity, perceived benefits, cues to action, self-efficacy and neck health-promoting behaviors in the intervention group increased significantly compared to the control group and the mean score of barriers. Perceived showed a significant decrease.

Findings from the present study showed that various factors such as age, physical activity, work experience and job satisfaction have been effective in teachers’ neck pain. These results were confirmed by the study of Patience N Erick et al. [29]. The findings of the study also showed that repetitive movements, inappropriate physical postures during activity and excessive use of force are the main factors in causing neck pain, the findings by the study of Maghsoudian et al. [12] and Cheng and Et al [24, 30] confirmed. The findings of the study also showed that contributing factors can play an effective role in the occurrence of behavior and facilitate the occurrence of the behavior, and their absence can prevent behavior change. These findings were confirmed by Goetsch DL study and colleagues [31].

The results of the evaluation of the educational intervention in the present study showed that the areas of awareness, perceived sensitivity, perceived intensity, perceived benefits, perceived barriers, Cues to Action, self-efficacy and behavior improved during the 3 months of follow-up in the intervention group. Educational intervention the present study was consistent with previous studies that stated that the use of the model in educational interventions can be effective in adopting health behaviors [32–34]. Increasing the average score of awareness in the intervention group is valuable, because having knowledge about neck pain, risk factors for this disease, as well as behaviors that promote neck health can create the right attitude about neck pain and adopt appropriate behavior [34]. In the present study, teachers’ awareness of neck pain increased for the intervention group during 3 months.

This finding was confirmed by the Janssens study [35]. Also, the results of the present study showed that the perceived barriers and benefits after educational intervention in the two groups are statistically significant. There was also a positive and significant relationship between perceived benefits and neck health-promoting behaviors. Increasing the score of perceived benefits after training, it is consistent with the results of Ghofranipour study [36]. In the present study, perceived sensitivity and severity, teachers’ self-efficacy in adopting neck health-promoting behaviors increased during the 3 months in the intervention group, these findings were confirmed by the study of Sharafkhani N and et al. [37] and study of Thompson R and et al. [38]. The present study showed that there is a positive correlation between self-efficacy and neck health-promoting behavior and higher self-efficacy indicates health behavior. This finding was confirmed by the study of Fung Seri et al. [34], and the study of Fida et al. [39]. The results show that managerial factors and organizational policies can play a very important role in adopting and promoting health behaviors. School administrators can equip the environment in terms of sports facilities, and spaces for teachers to rest and control stress at work.

A study by Ross et al. [40] confirms this finding. In the present study, the intervention based on social media was very successful. Social media facilitates user interaction and expands knowledge because it removes barriers to geographical distance and physical presence. The results of several studies confirm these finding [18, 41–43]. The results of the present study show the positive effect of the educational program designed based on the health belief model in the context of social networks, increasing perceived sensitivity, perceived severity, perceived benefits, Cues to Action and self-efficacy and reducing perceived barriers in the intervention group, this is followed by an increase in neck health-promoting behaviors in teachers. These findings indicate the effectiveness of educational intervention in adopting behaviors that promote neck health, prevention and reduction of neck pain.

### Study strengths and weaknesses

One of the most important strengths of the present study is the lack of similar studies in Iranian teachers. Other strengths include a combination of qualitative study and clinical trial, as well as the specific design and implementation of educational intervention and He mentioned the use of social media to provide educational content. As well, the presence of male and female participants and evaluating the effect of educational intervention on the adoption of neck health-promoting behaviors in both sexes were other strengths of the present study. It is also possible to follow the effect of the intervention 3 months after the intervention on the continuation of promotional behaviors as strengths of the study. One of the limitations of the present study was the teachers’ self-report on the severity of neck pain and recommended health behaviors. Also, another limitation of the study was the selection of teachers from the first high school of public schools in Tehran. This is because the views of these teachers, as well as the severity of the neck pain and the impact of the educational intervention, may be different from those of teachers in other grades, cities, and non-governmental schools. Given that sampling was selected as a call, the research team, after collecting the samples, contacted all the sample members by phone (based on a pre-designed structured interview based on the inclusion/ exclusion criteria) and selected the people who were eligible to participate in the study and other members were removed from the group. However, it was possible that the participants should not represent the target community that is a kind of probably limitation of this study. Thus it is recommended that the future studied design further researches without this limitation.

### Suggestions

Since the present study was conducted in the master’s degree, due to lack of time, 6-month and 1-year follow-ups were not possible, so it is suggested that in future studies, the long-term follow-up to investigate the effect of the intervention on the continuation of behavior. It is also suggested to study and compare the effect of educational intervention based on health belief model in adopting neck pain prevention behaviors in teachers of different grades (preschool, primary and secondary school).

## Data Availability

The data will be available from the corresponding author on request.
